# Supplementary material for: The association between epicardial adipose tissue thickness and diabetes mellitus, hyperlipidemia, hepatosteatosis, pancreatic steatosis and pancreatitis
Source: PLoS One. 2026 Feb 26;21(2):e0343831. doi: 10.1371/journal.pone.0343831 (PMC12944748; doi:10.1371/journal.pone.0343831)
Supplement: Attachment — Submitted filename: Response to Reviewers.docx [file pone.0343831.s001.docx]

Dear Editor,

We would like to thank you for your careful evaluation of our revised manuscript and for outlining the additional requirements necessary before further processing. We have addressed each point in detail below.

1) We confirm that the manuscript has been revised to fully comply with PLOS ONE’s style and formatting requirements, including file naming conventions. The revised files have been prepared using the official PLOS ONE templates for both the main text and the title/authors/affiliations page.

2) Regarding data availability, the study was conducted in a hospital affiliated with the Turkish Ministry of Health, and data sharing is subject to national policies governing patient data confidentiality and institutional data ownership. Although the dataset was de-identified for analysis, public data sharing is not permitted under Ministry of Health regulations. These restrictions were imposed at the institutional and regulatory level. Data access requests may be directed to the local Institutional Ethics Committee that approved the study. Accordingly, the Data Availability Statement has been updated to reflect that de-identified data are available upon reasonable request and with appropriate approvals, but cannot be deposited in a public repository.

3) We confirm that the corresponding author has an ORCID iD and will ensure that it is validated and linked in the Editorial Manager system prior to final acceptance, in accordance with PLOS ONE requirements.

Finally, all reviewer-suggested references have been carefully reviewed and evaluated. Relevant publications have been cited where appropriate, consistent with the editor’s guidance.

We believe that these revisions and clarifications fully address the editorial requirements. We thank you again for your guidance and consideration of our manuscript.

Sincerely,

Şehnaz EVRİMLER

On behalf of all authors

**RESPONSE TO REVIEWERS**

**Reviewer 1.**

**Point 1. I found the introduction rather weak in terms of focus and logical flow. You mention several conditions (hepatosteatosis, diabetes, pancreatitis, subcutaneous fat), but, there is no clear narrative connecting these elements. It feels as if several conditions were listed without a central rationale explaining why they were all included in the same study... The link between EAT and pancreatitis, in particular, seems weak and needs stronger biological justification. Since EAT is a cardiac fat depot, the biological pathway by which it could influence pancreatic inflammation is not evident. You might consider citing mechanistic or imaging studies, if available, or explaining your hypothesis more clearly. Also, stating that ""the link is not conclusive"" doesn’t clearly define the research gap. Readers need to understand waht exactely is uncertain or conflicting in the literature. I also noticed you didn’t refer to any existing studies with similar objectives or findings. Without that, it’s hard to see how your work differs from what’s already been done or what new aspect it brings. I suggest you restructure this section to highlight one clear gap, explain why these associations are relevant, and end with a precise hypothesis connecting EAT to the selected metabolic factors.**

Thank you very much for this insightful and constructive feedback. In line with your suggestions, we substantially revised the final paragraph of the Introduction to improve coherence, strengthen the biological rationale, and clearly articulate the research gap. First, we clarified that although EAT is anatomically a cardiac fat depot, it acts as an active visceral adipose tissue with systemic inflammatory effects and a strong correlation with total visceral fat, making its potential association with hepatic and pancreatic fat biologically plausible. We also added mechanistic justification by referencing the proinflammatory cytokines secreted by EAT and explaining how ectopic fat depots—including hepatic and pancreatic fat—share common metabolic and inflammatory pathways. Second, we specified that the relationships between EAT and pancreatic fat or pancreatitis history have not been systematically examined, and that existing literature on EAT and hepatosteatosis provides inconsistent results, thereby defining the precise gap our study addresses. Finally, we added a clearer rationale for why these metabolic factors were included together and concluded the Introduction with a precise, hypothesis-driven statement. All revisions have been incorporated into the manuscript and marked accordingly (Lines 61-73).

**Point 2: I think the methods section needs much more detail. While you report sample size and sex in the results section, it’s important to include this information in methods along with how participants were selected and the inclusion//exclusion criteria. Without that, it’s hard to know if your results are reliable or representative. Please consider adding these details so we can understand the conditions of your study and how representative your population is. Also, it would also help to clarify whether potential confounders like age, sex, or BMI were considered, as this is important for interpreting your results.**

Thank you very much for this valuable comment. We have substantially revised the Methods section to address your concerns and improve clarity and reproducibility. Specifically, we added detailed descriptions of the study population, including how patients were selected, the time frame of imaging, and the inclusion of abdominal CT/MRI examinations performed for suspected pancreatitis. We also incorporated explicit inclusion and exclusion criteria and clarified that age, sex, and BMI were collected as potential confounders relevant to ectopic fat distribution. In addition, we expanded the descriptions of the imaging measurements for EAT thickness, hepatosteatosis, and pancreatic fat, and clarified that all assessments were performed independently by two radiologists. These revisions ensure a complete and transparent methodological framework. All changes have been incorporated into the manuscript and marked accordingly (Lines 83-90 and 104-106).

**Point 3 : The results section is detailed, but it feels somewhat descriptive and difficult to follow in relation to your study objectives. You report many associations, yet it’s not always clear which ones are most central to your objective. I suggest reorganizing the results according to your main aims (for example, EAT and diabetes, EAT and pancreatitis, etc.) and adding short linking sentences to guide the reader through the key findings. This would make the section clearer and more coherent.**

Thank you for this valuable comment. We have substantially reorganized the Results section to improve clarity and alignment with our study aims. The findings are now presented under clear thematic subsections (e.g., EAT and Diabetes Mellitus, EAT and Hepatic Steatosis, EAT and Pancreatitis History), and brief linking sentences were added to guide the reader through the key results. These revisions improve the overall readability and coherence of the Results section. All changes have been incorporated into the revised manuscript (Lines 134-204).

**Point 4: I see that several of your results are statistically significant, but when I look at the actual correlation values. For example, between age, EAT, and subcutaneous fat (r = 0.15–0.32). These relationships appear quite weak. Even with low p values, such small effect sizes may not have much clinical meaning. I think it would really strengthen your paper if you could comment on whether these findings are meaningful in practice, or if they might simply reflect confounding factors like BMI or general adiposity.**

Thank you for this insightful observation. We agree that although several correlations reached statistical significance, their effect sizes were small (r = 0.15–0.32). To address this, we expanded the Discussion to clarify that these weak associations may have limited clinical relevance. We also added commentary noting that unmeasured confounders—particularly BMI and overall adiposity—may partially explain these relationships. The revised text now emphasizes the need for cautious interpretation and highlights this as an important limitation. These additions have been incorporated into the updated manuscript (Lines 257-263).

**Point 5: I noticed that some important potential confounders, such as BMI, overall body fat, hypertension, or medication use, don’t seem to have been accounted for in your analyses. Since EAT and subcutaneous fat are strongly influenced by these factors, the associations you report with diabetes, pancreatitis, or LDL cholesterol could be affected. Could you clarify whether these variables were available, and if so, consider including them in your multivariate models??? If not, a discussion of the possible impact of unmeasured confounding would help strengthen the study.**

Thank you for this insightful comment. Unfortunately, BMI, total body fat, hypertension status, and detailed medication history were not available in the retrospective dataset. We fully agree that these variables are important determinants of EAT and subcutaneous adiposity, and their absence may have introduced unmeasured confounding. To address this concern, we added a paragraph in the Discussion acknowledging this limitation and clarifying that the associations observed—particularly with diabetes, LDL cholesterol, and pancreatitis—should be interpreted with caution. We also highlighted the need for future prospective studies including comprehensive metabolic data (Lines 283- 287 and 293-301).

**Point 6: In the regression analyses, it’s not clear how you decided which variables to include in the multivariate models. Were variables selected based on univariate significance (e.g.,, p < 0.1), or were all measured variables considered???**

Thank you for this important comment. In our analysis, all measured variables were first entered into the univariate logistic regression. Only variables that showed statistical significance in the univariate analysis (p < 0.05) were subsequently included in the multivariate model. This stepwise approach was chosen to avoid overfitting and to ensure that only meaningful predictors were retained. Age and sex were kept in the model as clinically essential covariates. We have clarified this procedure in the Methods section (Lines 127-131).

**Point 7: I see that you mention the retrospective design as a limitation, which is good. However, some of the language in the results and discussion, such as "independent predictors"" implies causality. Since causality cannot be established in a retrospective study, I suggest clarifying this in the text and using more neutral language (e.g.. "variables significantly associated with…") to avoid overinterpretation.**

Thank you for this important comment. We fully agree that causal interpretations cannot be made in a retrospective study. In accordance with your suggestion, we carefully reviewed the Results and Discussion sections and revised the wording to avoid causal language. Terms such as “independent predictor” were replaced with neutral expressions including “significantly associated with” reflecting statistical relationships rather than causality. These revisions ensure that the findings are interpreted appropriately within the limitations of the study design (Lines 32-39, 162, 213-217).

**Point8: The prevalence of pancreatitis (73.5%%) and diabetes (31.4%) in your cohort seems unusually high compared with the general population. Could this reflect a selection bias; for example, if the sample mainly includes patients undergoing CT for pancreatic evaluation?? Could you clarify your sampling source and whether this distribution is representative of the broader population you aim to study..**

Thank you for this important observation. The high prevalence of pancreatitis and diabetes in our cohort indeed reflects a targeted sampling strategy rather than the general population distribution. As one of the predefined aims of the study was to examine the relationship between EAT and pancreatitis, patients who underwent abdominal CT/MRI due to clinical suspicion of pancreatitis were intentionally included. Therefore, the sample is enriched with individuals evaluated for possible pancreatitis, which explains the higher prevalence. We have clarified this point in the Methods section to avoid misinterpretation (Lines 83-86).

**Reviewer 2.**

**Point 1. The abstract lacks a clear problem statement as it lists results but does not sufficiently justify why this research matters or what specific knowledge gap it addresses. The final sentence should highlight the broader relevance rather than repeat outcomes.**

Thank you for your comment. We revised the abstract to clearly state the knowledge gap regarding the unclear relationship between EAT, pancreatic steatosis, and pancreatitis, and to justify the clinical relevance of the study. We also adjusted the final sentence to emphasize the broader significance rather than repeating results. We believe these changes enhance clarity and focus (Lines 18-21, 32-33, 35-39).

**Point 2. The research gap is weakly articulated in the introduction. While prior work is mentioned, the authors do not convincingly explain what was missing in existing literature that this study uniquely addresses.**

Thank you for this insightful comment. We revised the Introduction to more clearly define the research gap. The updated text now explicitly highlights: the lack of studies examining the relationship between EAT and pancreatic fat or pancreatitis, inconsistent evidence regarding the association between EAT and hepatosteatosis, and the uncertainty about whether EAT reflects only cardiac adiposity or broader systemic ectopic fat accumulation. These revisions strengthen the rationale and clarify the unmet need addressed by our study (Lines 61-73).

**Point 3. The rationale for selecting the study design is limited. The manuscript does not explain why the chosen population, tools, and parameters are most appropriate to answer the stated research question.**

Thank you for the comment. We clarified in the Methods why this study population and imaging parameters were appropriate. Abdominal CT/MRI enabled simultaneous evaluation of EAT, hepatic steatosis, pancreatic fat, and pancreatitis, directly aligning with our research aims. We also specified that patients imaged for suspected pancreatitis were intentionally included because assessing the EAT–pancreatitis relationship was one of the study objectives (Lines 83-86).

**Point 4. Methodology lacks detail and reproducibility in some areas. Key procedural specifics (e.g., selection criteria, sample handling, or validation of measurement tools) are missing or insufficiently described. This may prevent replication.**

Thank you for your constructive comment. We expanded the Methods section to provide full reproducibility, including detailed inclusion/exclusion criteria, rationale for the study population, CT/MRI measurement protocols and HU-based definitions (Lines 86-90).

**Point 5. The study seems observational/descriptive, yet some statements imply causal relationships. These need to be toned down or reframed as associations only.**

Thank you for this important remark. We carefully revised the manuscript to remove any causal wording. All expressions suggesting prediction or causality were replaced with neutral, association-based terminology (e.g., “significantly associated with”). The revised text now accurately reflects the observational nature of the study (Lines 163, 194-195, 216-217, 248-252).

**Point 6. Sample size justification is not clearly provided. A power analysis or rationale for participant numbers would strengthen the manuscript.**

Thank you for your constructive comment. Because this was an exploratory, retrospective study, no formal a priori sample size calculation was performed. The sample size was determined by the number of consecutive patients who met the inclusion criteria during the study period, which we considered sufficient to explore the hypothesized associations; however, future prospective studies with formal power calculations are warranted.

**Point 7. The statistical analysis section is too brief. The authors should clarify corrections for multiple testing, how missing data were handled, and whether data met normality assumptions before applying parametric tests.**

We thank the reviewer for this comment. There were no missing data in the dataset. Normality assumptions were assessed prior to applying parametric tests, and the relevant details have now been clarified and explicitly stated in the statistical analysis section (Lines 118-132).

**Point 8. Results presentation feels fragmented. While data are provided, there is limited integration of findings into a coherent narrative. Tables/figures could be better linked in the text with clearer interpretation rather than simple reporting.**

Thank you for this valuable comment. We have revised the Results section to create a more coherent and integrated narrative. Data are now presented with clearer transitions, and tables are referenced at points directly relevant to their findings. Each table is accompanied by brief interpretative statements rather than simple numerical reporting (Lines 133-204).

**Point 9. Tables and figures appear overly descriptive. Several contain too much raw numerical detail without clear summaries or take-home messages. They also need clearer captions that explain what readers should focus on.**

Thank you for this helpful comment. In line with your suggestion, we revised the caption of Table 1 to clearly highlight its key findings. Additionally, the captions of all other tables were rewritten to be more explanatory and to guide the reader toward the main take-home messages. These revisions improve clarity, reduce excessive descriptive detail, and enhance the interpretability of the presented results (Lines 153, 164, 179, 196, 203).

**Point 10. Discussion largely restates results rather than interpreting them. There is little critical reflection on why certain outcomes emerged, how they compare to similar studies, or what mechanisms may be involved.**

We thank the reviewer for this comment. The Discussion section has been revised to reduce repetition of the Results and to provide more interpretative commentary. Specifically, we added critical reflections on the potential reasons underlying the observed associations, compared our findings with relevant prior studies, and discussed possible biological and inflammatory mechanisms where appropriate (Lines 209-301).

**Point 11. Limitations are acknowledged but underdeveloped. Key issues such as potential confounders, selection bias, and measurement limitations deserve a more honest discussion.**

We thank the reviewer for this comment. The Limitations section has been expanded to more explicitly address potential selection bias, unmeasured confounders, and measurement-related limitations, including the retrospective single-center design, inclusion of patients with suspected pancreatitis, absence of key metabolic variables, and imaging-based assessment of EAT and steatosis, noting that volumetric segmentation of EAT was not performed and that measurements were based on averaged thickness values rather than full volumetric analysis. We believe these revisions provide a more transparent and balanced interpretation of the findings (Lines 283-287).

**Point 12. The manuscript overextends its conclusions. The findings support limited association but not practical recommendations. The authors should temper claims to match the strength of their data.**

We thank the reviewer for this comment. We have revised the Conclusion to temper the language and ensure that the claims are aligned with the observational nature and strength of the data, removing terms implying strong correlations, emphasizing associations rather than practical recommendations and highlighting the need for future prospective studies (Lines 305-308).

**Point 13. The implications for policy, clinical relevance, or future research are not clearly drawn. The conclusion could be more forward-looking, stating how these results should guide subsequent work.**

We thank the reviewer for this suggestion. We have revised the Conclusion to be more forward-looking by explicitly outlining how the findings should guide future prospective and mechanistic studies, rather than immediate clinical application (Lines 311-314).

**Point 14. Language is generally understandable, but certain paragraphs feel stiff and overly technical, reducing flow. Some grammatical issues remain; the manuscript would benefit from careful editing for clarity and conciseness.**

We thank the reviewer for this comment. The manuscript has been carefully edited for clarity, conciseness, and improved flow. Stiff or overly technical phrasing has been simplified, and remaining grammatical issues have been corrected throughout the text.

**Point 15. Overall novelty appears modest. The study adds incremental information rather than a fundamentally new insight. The authors should emphasize what is genuinely new i.e., population studied, new analytical approach, or unique findings if applicable.**

We thank the reviewer for this comment. We have revised the Discussion to more clearly emphasize the incremental novelty of the study, particularly the evaluation of epicardial adipose tissue thickness on CT scans obtained for suspected pancreatitis, a population that has been less frequently examined in prior EAT research. We believe this clarification highlights the specific and genuine contribution of the present work (Lines 213-215).
